# Health Care Utilization Patterns Among Adults With or Without Functional Disabilities

**DOI:** 10.1001/jamanetworkopen.2025.4729

**Published:** 2025-04-11

**Authors:** Sungchul Park, Jim P. Stimpson, A. Mark Fendrick

**Affiliations:** 1 BK21 FOUR R&E Center for Learning Health Systems, Department of Health Policy and Management, College of Health Science, Korea University, Seoul, Republic of Korea; 2 Department of Health Economics, Systems, and Policy, Peter O’Donnell Jr School of Public Health, The University of Texas Southwestern Medical Center, Dallas; 3 Department of Internal Medicine, School of Medicine, University of Michigan, Ann Arbor; 4 Department of Health Management and Policy, School of Public Health, University of Michigan, Ann Arbor; 5 Center for Value-Based Insurance Design, University of Michigan, Ann Arbor

## Abstract

**Importance:**

Adults with functional disabilities require more medical care, but it remains unclear whether they use more health services, including high- and low-value services.

**Objectives:**

To examine health care utilization by functional disability among US adults.

**Design, Setting, and Participants:**

This cross-sectional study analyzed data from the 2013 to 2021 Medical Expenditure Panel Survey. The sample comprised noninstitutionalized US civilians aged 18 years or older. Statistical analysis was conducted between May and October 2024.

**Exposures:**

Self-reported functional disability. Functional disability was assessed through 6 questions on difficulties (with vision, hearing, memory or concentration, walking, self-care, and performing errands) and categorized as no (0 difficulties), moderate (1-2 difficulties), and severe (≥3 difficulties).

**Main Outcomes and Measures:**

Outpatient visits, prescription drug fills, 10 high-value services, and 12 low-value services.

**Results:**

The sample comprised 188 954 adults (mean [SD] age, 48.1 [17.9] years; 101 706 females [53.8%]). Of these adults, 151 562 (80.2%) had no, 28 518 (15.0%) had moderate, and 8874 (4.6%) had severe functional disabilities. Adults with functional disabilities, especially those with severe disabilities, had a higher percentage of outpatient visits (86.2% vs 74.9%) and prescriptions filled (81.2% vs 64.2%) compared with those with no disabilities. The mean number of outpatient visits and prescription drug fills was significantly higher among those with severe vs no or moderate functional disabilities (outpatient visits: 17.5 [95% CI, 16.5-18.4] vs 8.6 [95% CI, 8.6-8.7] or 14.0 [95% CI, 13.8-14.3]; prescription drug fills: 27.8 [95% CI, 25.7-29.9] vs 10.6 [95% CI, 10.5-10.7] or 18.0 [95% CI, 17.6-18.4], respectively). Compared with adults with no functional disabilities, those with moderate and severe disabilities had higher rates of services that could be performed during an appointment, both high value (eg, adjusted differences, blood pressure measurement: 3.4 [95% CI, 2.9-3.9] percentage points and 3.6 [95% CI, 2.9-4.2] percentage points; cholesterol measurement: 3.6 [95% CI, 2.6-4.5] percentage points and 4.7 [95% CI, 3.6-5.7] percentage points, respectively) and low value (eg, adjusted differences, benzodiazepine for depression: 4.5 [95% CI, 2.5-6.4] percentage points and 8.1 [95% CI, 6.3-9.8] percentage points; opioid for back pain: 4.5 [95% CI, 3.5-5.5] percentage points and 6.7 [95% CI, 6.5-6.9] percentage points, respectively). Conversely, those with moderate and severe disabilities used fewer services that typically required a separate appointment, such as high-value cancer screenings (eg, adjusted differences, breast: −1.1 [95% CI, −1.3 to −0.9] percentage points and −9.9 [95% CI, −12.1 to −7.7] percentage points; cervical: −3.3 [95% CI, −4.9 to −1.7] percentage points and −17.3 [95% CI, −20.3 to −14.4] percentage points, respectively) and low-value cancer screenings (eg, adjusted differences, cervical: −4.9 [95% CI, −7.7 to −2.1] percentage points and −8.1 [95% CI, −12.1 to −4.0] percentage points, respectively).

**Conclusions and Relevance:**

In this cross-sectional study, adults with functional disabilities used higher rates of health services than adults with no functional disabilities. However, the ease of access to services—independent of clinical value—plays an important role in utilization for those with functional disabilities.

## Key Points

**Question:**

Do adults with self-reported functional disabilities use high- and low-value services at different rates compared with adults without functional disabilities?

**Findings:**

In this cross-sectional study of 188 954 US adults, those with functional disabilities had higher rates of outpatient visits and prescription drug fills than adults with no disabilities, but utilization of high- and low-value services varied. Readily accessible services (eg, diagnostic tests and medications) were used more frequently by adults with disabilities, while services requiring separate appointments (eg, cancer screenings) were used less often.

**Meaning:**

The findings suggest that accessibility to health services regardless of clinical value plays a critical role in utilization for adults with functional disabilities.

## Teaser

This cross-sectional study examines the association between self-assessed difficulty in performing daily tasks and rates of outpatient visits, prescription drug fills, and use of other health services among adults.

## Introduction

Functional disability, defined as difficulty in performing daily tasks or maintaining independent living, is common in the US. In 2022, approximately 1 in 4 US adults reported experiencing some form of disability.^1^ The most prevalent types included cognitive disabilities, followed by mobility, independent living, hearing, vision, and self-care disabilities. These disabilities can play a direct role in acute health issues or an indirect role in the care provided for nondisabling conditions and ultimately in quality of life and health status.^2^

Adults with functional disabilities often face a complex combination of medical, behavioral health, and social challenges,^3^ which are associated with higher health care utilization.^4,5,6,7,8^ Specifically, the onset of activity limitation is associated with a 4-fold increase in emergency department visits and hospitalizations.^9^ Additionally, adults with functional disabilities use home health services and equipment and supplies at higher rates than those without functional disabilities.^4^ Consequently, health care expenditures for adults with functional disabilities exceed those for adults without functional disabilities, increasing disproportionately with the severity of the disability and ranging from $1934 for those needing no assistance with activities of daily living (ADL) to $14 399 for those needing assistance with 5 to 6 ADLs.^7^

However, higher health care utilization may not necessarily lead to higher quality, equity, and efficiency of care delivery.^10^ In 2019, annual spending on unnecessary, and sometimes harmful, care ranged from $75.7 billion to $101.2 billion.^11^ This phenomenon may be more prevalent among vulnerable populations. Specifically, individuals from racial and ethnic minority groups and individuals with lower income or educational levels are less likely to use high-value care and more likely to receive low-value care.^12,13,14^ Among these socioeconomically vulnerable groups, individuals with functional disabilities are particularly at risk due to greater needs for health care.^15^ However, this population often has lower health literacy, which may be associated with a reduced understanding of high- and low-value care. While prior studies have shown that adults with functional disabilities generally use more health services in total than adults without disabilities,^4,5,6,7,8^ it remains unclear whether this population uses high- and low-value services at different rates and what service characteristics might explain these variations.

To examine health care utilization by functional disability, we conducted 2 main analyses. First, we compared health care utilization and unmet need for medical care among US adults with varying levels of functional disability (no, moderate, or severe). We hypothesized that adults with functional disabilities have more outpatient visits, fill more prescriptions, and report greater unmet need for medical care compared with adults with no functional disabilities. Second, we assessed whether the case provided was supported by evidence of clinical benefits—that is, the use of high- and low-value services across the 3 levels of functional disability. We hypothesized that utilization patterns of high- and low-value services are inconsistent among adults with functional disabilities, but ease of access plays a key role in service utilization for this population.

## Methods

We used data from the 2013 to 2021 Medical Expenditure Panel Survey (MEPS), a nationally representative survey of the US civilian noninstitutionalized population (eMethods in Supplement 1).^16^ The sample for this cross-sectional study consisted of US adults (aged ≥18 years). The institutional review board at Korea University deemed this study exempt from ethics review and the informed consent requirement because it used publicly available, deidentified data. We followed the Strengthening the Reporting of Observational Studies in Epidemiology (STROBE) reporting guideline.

### Primary Independent Variable

The primary independent variable was self-reported functional disability, which refers to limitations or difficulties in performing everyday activities due to physical, mental, or emotional conditions. Based on prior research,^4,17^ functional disability was ascertained using 6 questions assessing difficulties in vision, hearing, memory or concentration, walking, self-care, and performing errands due to a physical, mental, or emotional condition. Questions from the MEPS were as follows: “Does anyone in the family have any difficulty seeing?”; “Does anyone in the family have any difficulty hearing?”; “Do any of the adults in the family experience confusion or memory loss such that it interferes with daily activities?”; “Does anyone in the family have any difficulty walking, climbing stairs, grasping objects, reaching overhead, lifting, bending or stooping, or standing for long periods?”; “Does anyone in the family receive help or supervision with personal care such as bathing, dressing, or getting around the house?”; and “Because of a physical, mental, or emotional condition, do you have difficulty doing errands alone such as visiting a doctor’s office or shopping?” If the response was yes to any of these questions, a follow-up question was asked to determine which household member had difficulty. These questions were originally developed and tested in the 1990s to identify disabling conditions and are now used in more than 10 national surveys conducted by various federal departments and agencies, including the US Census Bureau.^18^ Furthermore, this 6-item set adheres to the standards for disability-related survey questions established by the US Department of Health and Human Services.^19^

We assigned a value of 1 to indicate the presence of a difficulty and 0 to indicate no difficulty, aggregating the scores from the 6 questions. Based on prior research,^4,17^ functional disability was classified into 3 levels: no (0 difficulties), moderate (1-2 difficulties), and severe (≥3 difficulties).

### Outcomes

We included 3 main outcome measures. First, we examined the utilization of 2 nonspecific health care service categories: outpatient visits and prescribed drug fills. These services were selected because they align directly with the settings in which we defined high- and low-value care. For each measure, we analyzed service utilization as a binary variable and the frequency of utilization as a continuous variable. As a secondary outcome, we investigated 2 binary self-reported measures of unmet need for medical care: delay in getting necessary medical care and inability to get medical care (eMethods in Supplement 1).

Second, we followed prior research using the MEPS^20,21,22,23^ to examine binary utilization measures of 10 specific high-value services across 3 categories: cancer screening (age-determined breast,^24^ cervical,^25^ and colorectal cancer screening^26^), diagnostic and preventive tests (dental checkup, blood pressure [BP] measurement,^27^ cholesterol measurement,^28^ and influenza vaccination^29^), and diabetes care (hemoglobin A_1c_ [HbA_1c_] measurement, foot examination, and eye examination^30^). Third, we investigated binary utilization measures of 12 specific low-value services across 3 categories: cancer screening (age-determined cervical,^25^ colorectal,^26^ and prostate cancer screening^31^ [eMethods in Supplement 1]), medication use (antibiotic for acute upper respiratory tract infection^32,33^; antibiotic for influenza^32^; benzodiazepine for depression^34^; opioid for back pain^35^; opioid for headache^36^; and nonsteroidal anti-inflammatory drug [NSAID] for hypertension, heart failure, or chronic kidney disease^34^), and imaging use (magnetic resonance imaging [MRI] or computed tomography [CT] for back pain, radiography for back pain, and MRI or CT for headache^37^).

For each measure, we identified those who were eligible for the measure (the denominator) using age, sex, and health conditions based on *International Classification of Diseases, Ninth Revision, Clinical Modification* and *International Statistical Classification of Diseases, Tenth Revision, Clinical Modification* diagnosis and procedure codes. We then determined whether eligible individuals received specific services (the numerator). Definitions for each outcome measure are presented in eTable 1 in Supplement 1.

### Statistical Analysis

To adjust for differences in sample characteristics, we included age, sex, self-reported race and ethnicity, employment status, marital status, educational level, family income, health insurance coverage, US Census region, and chronic conditions (eTable 2 in Supplement 1). We calculated sample characteristics by functional disability levels. To quantify differences in outcomes by functional disability levels, we used a logistic regression model for binary outcomes and a linear regression model for continuous outcomes after controlling for individual-level characteristics and year fixed effects. Using the marginal effects from these models, we estimated the mean adjusted values of the outcomes for each group while holding all other variables constant except the variable of interest. Furthermore, we estimated the adjusted differences in the outcomes between adults with severe and moderate functional disabilities relative to those with no functional disabilities.

For all analyses, we clustered SEs within individuals, as some individuals were included in the data over the course of multiple years. We used survey weights to generate nationally representative estimates. Data analysis was performed between May and October 2024 using Stata, version 17.0 (StataCorp LLC).

## Results

### Sample Characteristics

The final sample included 188 954 adults (mean [SD] age, 48.1 [17.9] years), of whom 101 706 were females (53.8%) and 87 248 were males (46.1%) and 11.5% reporting as Hispanic, 3.6% as non-Hispanic Asian, 15.2% as non-Hispanic Black, 67.1% as non-Hispanic White, and 2.6% as non-Hispanic other or multiracial individuals. Of these adults, 156 818 (80.4%) had no functional disabilities, 29 101 (14.9%) had moderate functional disabilities, and 9058 (4.6%) had severe functional disabilities (Table 1).

**Table 1.** Sample Characteristics of US Adults by Functional Disability

Characteristic	Adults, No. (weighted %) (N = 188 954)^a^^,^^b^		
	No functional disability (n = 151 562 [80.4%])	Moderate functional disability (n = 28 518 [14.9%])	Severe functional disability (n = 8874 [4.6%])
Age group, y			
18-24	16 996 (11.6)	1183 (4.7)	148 (2.1)
25-44	29 918 (20.7)	2031 (7.6)	297 (3.3)
45-64	29 447 (18.7)	2476 (8.2)	531 (5.5)
65-74	27 172 (17.9)	3890 (13.0)	1167 (12.2)
≥75	23 957 (16.2)	6189 (21.1)	1809 (19.0)
Sex			
Male	71 411 (49.0)	12 557 (46.7)	3280 (38.9)
Female	80 151 (51.0)	15 961 (53.3)	5594 (61.1)
Race and ethnicity, self-reported			
Hispanic	40 552 (17.1)	4740 (10.6)	1720 (11.5)
Non-Hispanic Asian	11 243 (6.6)	905 (2.7)	279 (3.1)
Non-Hispanic Black	24 708 (11.8)	5286 (11.3)	1766 (13.0)
Non-Hispanic White	71 080 (61.8)	16 570 (71.7)	4740 (68.5)
Non-Hispanic other or multiple^c^	3979 (2.7)	1017 (3.8)	369 (4.0)
Employed	10 1258 (70.7)	8904 (34.9)	655 (8.4)
Married	78 324 (54.8)	11 981 (46.6)	2728 (35.0)
Educational level			
No high school diploma	23 334 (9.9)	5780 (14.9)	2672 (23.7)
High school diploma	61 506 (40.0)	13 376 (47.4)	4033 (47.7)
≥College degree	66 722 (50.0)	9362 (37.7)	2169 (28.6)
Family income, % of FPL			
<200	50 117 (23.6)	13 690 (39.2)	5594 (54.7)
200-399	44 834 (28.9)	7673 (27.6)	2030 (25.5)
≥400	56 611 (47.4)	7155 (33.2)	1250 (19.8)
Health insurance coverage			
Any	130 387 (90.0)	26 722 (94.7)	8597 (97.1)
Medicaid	20 287 (9.6)	7126 (18.9)	3784 (34.8)
Medicare	25 099 (15.5)	15 451 (53.7)	6401 (73.5)
Private	87 913 (66.1)	7371 (30.1)	763 (10.1)
US Census region			
Northeast	24 527 (17.9)	4619 (16.5)	1467 (17.0)
Midwest	28 713 (20.6)	6221 (22.8)	1795 (21.6)
South	56 284 (37.2)	11 309 (39.7)	3683 (41.0)
West	42 038 (24.2)	6369 (20.9)	1929 (20.5)
No. of chronic conditions			
0	18 307 (12.9)	6475 (23.5)	1859 (20.7)
1-2	16 680 (10.6)	8456 (29.1)	3309 (37.4)
3-5	1913 (1.2)	2434 (8.3)	1741 (18.9)
≥6	32 (0.0)	152 (0.6)	185 (2.2)

Abbreviation: FPL, federal poverty level.

^a^ Functional disability was measured using 6 questions assessing difficulties and was categorized into 3 levels: no (0 difficulties), moderate (1-2 difficulties), and severe (≥3 difficulties).

^b^ Survey weights were used to adjust sample characteristics to be representative of the US population.

^c^ In the Medical Expenditure Panel Survey, other or multiple race and ethnicity typically includes those who did not identify with 1 of the primary categories (Hispanic, non-Hispanic Asian, non-Hispanic Black, or non-Hispanic White) or identified as multiracial.

We found notable differences in sample characteristics based on functional disability levels. Compared with adults with no functional disabilities, adults with functional disabilities were more likely to be older, be non-Hispanic White individuals, have Medicaid or Medicare coverage, and have more chronic medical conditions and were less likely to be employed, married, have a college graduate degree or higher, have higher family income, and have private insurance coverage. These differences were especially pronounced among adults with severe functional disabilities.

### Outpatient Visits and Prescription Drug Fills

**Figure 1.**
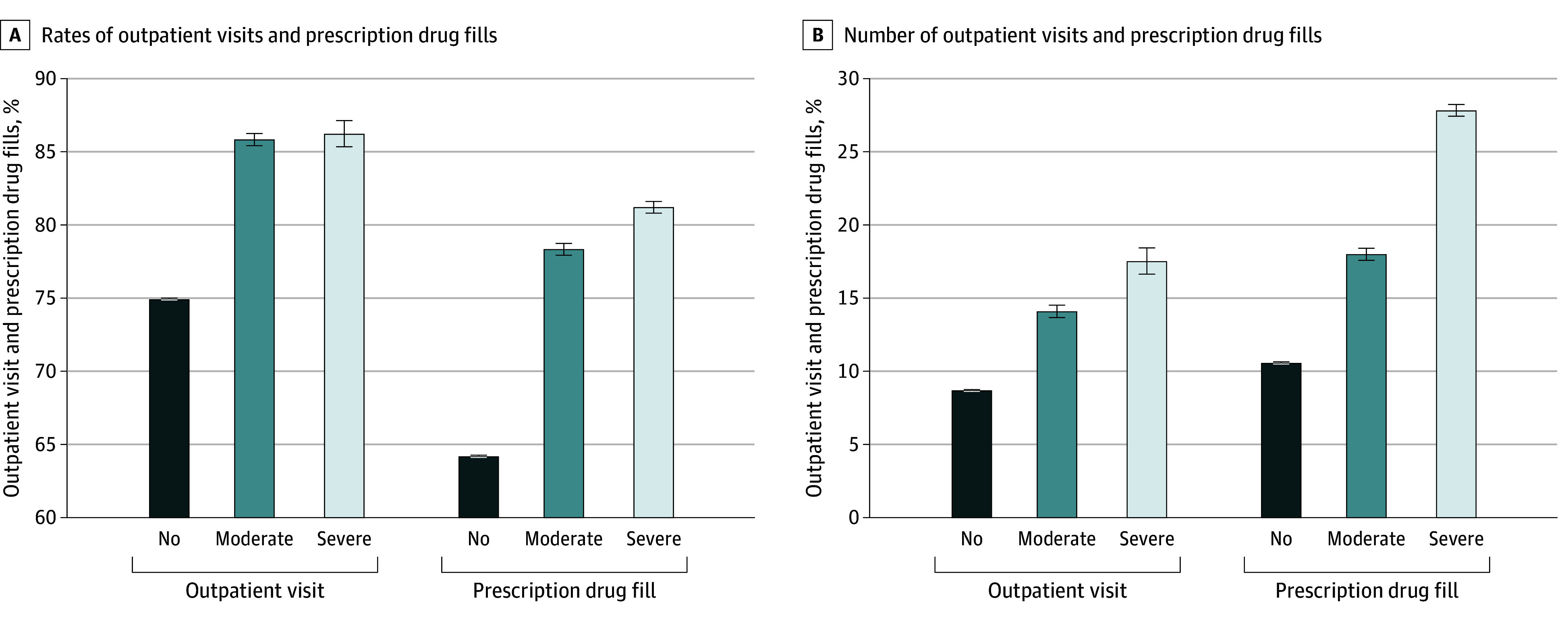
Outpatient Visits and Prescription Drug Fills Among US Adults by Functional Disability Error bars represent 95% CIs. Logistic regression models were used to estimate adjusted differences in the outcomes, controlling for individual-level characteristics (age, sex, self-reported race and ethnicity, employment status, marital status, educational level, family income, health insurance coverage, US Census region, and chronic conditions) and year fixed effects. Survey weights were used to generate nationally representative estimates of adults from the 2013 to 2021 Medical Expenditure Panel Survey.

When evaluating service use as a binary outcome, we found that compared with adults with no functional disabilities, those with moderate and severe functional disabilities had higher rates of outpatient visits and prescription drug fills. Specifically, outpatient visit rates were 74.9% (95% CI, 74.7%-75.0%) for adults with no functional disabilities, 85.8% (95% CI, 84.8%-86.8%) for adults with moderate functional disabilities, and 86.2% (95% CI, 84.1%-88.3%) for adults with severe functional disabilities (Figure 1). Prescription drug fill rates were 64.2% (95% CI, 64.0%-64.3%) for adults with no functional disabilities compared with 78.3% (95% CI, 77.5%-79.1%) for adults with moderate and 81.2% (95% CI, 80.4%-82.0%) for those with severe functional disabilities.

When evaluating service use as a continuous outcome, we observed more pronounced differences in utilization between those with moderate and severe functional disabilities. The mean number of outpatient visits and prescription drug fills was significantly higher among those with severe vs no or moderate functional disabilities (outpatient visits: 17.5 [95% CI, 16.5-18.4] vs 8.6 [95% CI, 8.6-8.7] or 14.0 [95% CI, 13.8-14.3], respectively; prescription drug fills: 27.8 [95% CI, 25.7-29.9] vs 10.6 [95% CI, 10.5-10.7] or 18.0 [95% CI, 17.6-18.4], respectively) (Figure 1).

### Unmet Need for Medical Care

**Figure 2.**
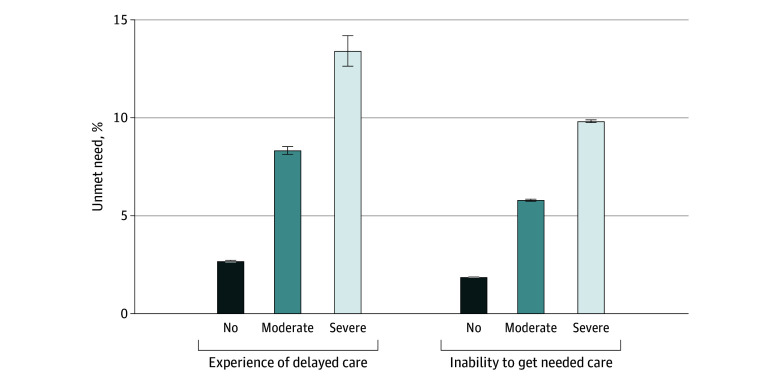
Unmet Need for Medical Care Among US Adults by Functional Disability Error bars represent 95% CIs. Logistic regression models were used to estimate adjusted differences in the outcomes, controlling for individual-level characteristics (age, sex, self-reported race and ethnicity, employment status, marital status, educational level, family income, health insurance coverage, US Census region, and chronic conditions) and year fixed effects. Survey weights were used to generate nationally representative estimates of adults from the 2013 to 2021 Medical Expenditure Panel Survey.

Unmet need for medical care was significantly higher among adults with functional disabilities, especially those with severe functional disabilities. Specifically, 8.3% (95% CI, 7.9%-8.7%) of adults with moderate functional disabilities and 13.4% (95% CI, 11.9%-14.9%) of adults with severe functional disabilities reported experiencing delays in getting necessary medical care compared with 2.7% (95% CI, 2.6%-2.8%) of adults with no functional disabilities (Figure 2). Furthermore, 5.8% (95% CI, 5.7%-5.9%) of adults with moderate functional disabilities and 9.8% (95% CI, 9.7%-9.9%) of those with severe functional disabilities reported being unable to get needed medical care compared with 1.9% (95% CI, 1.8%-1.9%) of adults with no functional disabilities.

### High-Value Care

The utilization of high-value care varied by service type and did not consistently increase with the severity of functional disability. Compared with adults with no functional disabilities, those with moderate and severe functional disabilities were more likely to receive certain high-value services, including BP measurement (adjusted differences: 3.4 [95% CI, 2.9-3.9] percentage points and 3.6 [95% CI, 2.9-4.2] percentage points, respectively), cholesterol measurement (adjusted differences: 3.6 [95% CI, 2.6-4.5] percentage points and 4.7 [95% CI, 3.6-5.7] percentage points, respectively), influenza vaccination (adjusted differences: 2.4 [95% CI, 2.1-2.8] percentage points for those with moderate functional disabilities), HbA_1c_ measurement (adjusted differences: 3.1 [95% CI, 1.2-5.1] percentage points and 3.8 [95% CI, 0.9-6.7] percentage points, respectively), and foot examination for diabetes care (adjusted differences: 5.8 [95% CI, 0.7-10.8] percentage points for those with severe functional disabilities) (Table 2). In contrast, those with moderate and severe functional disabilities were less likely to receive other high-value services, including breast cancer screening (adjusted differences: −1.1 [95% CI, −1.3 to −0.9] percentage points and −9.9 [95% CI, −12.1 to −7.7] percentage points, respectively), cervical cancer screening (adjusted differences: −3.3 [95% CI, −4.9 to −1.7] percentage points and −17.3 [95% CI, −20.3 to −14.4] percentage points, respectively), and dental checkups (adjusted differences: −2.0 [95% CI, −2.5 to −1.4] percentage points and −8.9 [95% CI, −10.4 to −7.4] percentage points, respectively).

**Table 2.** Use of High-Value Care Among US Adults by Functional Disability

High-value care service	Functional disability^a^^,^^b^						Adjusted differences vs no functional disability (95% CI), percentage point^c^	
	No		Moderate		Severe			
	Eligible sample, No.	Recipient, No. (%)	Eligible sample, No.	Recipient, No. (%)	Eligible sample, No.	Recipient, No. (%)	Moderate functional disability	Severe functional disability
Cancer screening								
Breast	2801	1675 (59.8)	1398	695 (49.7)	614	196 (31.9)	−1.1 (−1.3 to −0.9)	−9.9 (−12.1 to −7.7)
Cervical	11 526	8402 (72.9)	3670	2708 (73.8)	1133	733 (68.2)	−3.3 (−4.9 to −1.7)	−17.3 (−20.3 to −14.4)
Colorectal	22 159	11 611 (52.4)	6693	3942 (58.9)	1922	1113 (57.9)	1.1 (−2.1 to 4.2)	−1.7 (−3.9 to 0.5)
Diagnostic and preventive tests								
Dental checkup	156 818	56 768 (36.2)	29 101	10 476 (36.0)	9058	2527 (27.9)	−2.0 (−2.5 to −1.4)	−8.9 (−10.4 to −7.4)
BP measurement	45 538	34 108 (74.9)	12 775	11 255 (88.1)	4400	4066 (92.4)	3.4 (2.9 to 3.9)	3.6 (2.9 to 4.2)
Cholesterol measurement	67 086	59 170 (88.2)	12 428	11 968 (96.3)	3750	3686 (98.3)	3.6 (2.6 to 4.5)	4.7 (3.6 to 5.7)
Influenza vaccination	27 408	14 937 (54.5)	10 997	7203 (65.5)	4011	2740 (68.3)	2.4 (2.1 to 2.8)	−0.3 (−1.7 to 1)
Diabetes care								
HbA_1c_ measurement	7223	5641 (78.1)	3609	3014 (83.5)	1482	1269 (85.6)	3.1 (1.2 to 5.1)	3.8 (0.9 to 6.7)
Foot examination	9403	5877 (62.5)	5000	3375 (67.5)	2189	1587 (72.5)	1 (−0.9 to 3.0)	5.8 (0.7 to 10.8)
Eye examination	9276	5612 (60.5)	4934	3192 (64.7)	2158	1409 (65.3)	−0.6 (−2.3 to 1.1)	0.4 (−2.0 to 2.8)

Abbreviations: BP, blood pressure; HbA_1c_, hemoglobin A_1c_.

^a^ Functional disability was measured using 6 questions assessing difficulties and was categorized into 3 levels: no (0 difficulties), moderate (1-2 difficulties), and severe (≥3 difficulties).

^b^ Survey weights were used to generate nationally representative estimates of adults from the 2013 to 2021 Medical Expenditure Panel Survey.

^c^ Logistic regression models were used to estimate adjusted differences in the outcomes, controlling for individual-level characteristics and year fixed effects.

High-value services that could be performed during an appointment, such as BP and cholesterol measurements, were more commonly used by adults with moderate and severe functional disabilities. In contrast, services typically requiring a separate appointment, such as breast and cervical cancer screenings, were used less often.

### Low-Value Care

Similarly, the utilization of low-value care varied by service type and did not show a consistent increase with the self-reported presence or severity of functional disability. Compared with adults with no functional disabilities, those with moderate and severe functional disabilities were more likely to receive certain low-value services, including benzodiazepine for depression (adjusted differences: 4.5 [95% CI, 2.5-6.4] percentage points and 8.1 [95% CI, 6.3-9.8] percentage points, respectively); opioid for back pain (adjusted differences: 4.5 [95% CI, 3.5-5.5] percentage points and 6.7 [95% CI, 6.5-6.9] percentage points, respectively); NSAID for hypertension, heart failure, or kidney disease (adjusted differences: 4.7 [95% CI, 3.7-5.7] percentage points and 3.4 [95% CI, 1.1-5.7] percentage points, respectively); MRI or CT for back pain (adjusted differences: 2.6 [95% CI, −0.1 to 5.4] percentage points and 5.8 [95% CI, 3.3-8.3] percentage points, respectively); and radiography for back pain (adjusted differences: 2.1 [95% CI, 1.3-2.9] percentage points and 4.3 [95% CI, 3.7-4.9] percentage points, respectively) (Table 3).

**Table 3.** Use of Low-Value Care Among US Adults by Functional Disability

Low-value care service	Functional disability^a^^,^^b^						Adjusted differences vs no functional disability (95% CI), percentage point^c^	
	No		Moderate		Severe			
	Eligible sample, No.	Recipient, No. (%)	Eligible sample, No.	Recipient, No. (%)	Eligible sample, No.	Recipient, No. (%)	Moderate functional disability	Severe functional disability
Cancer screening								
Cervical	4019	985 (24.5)	2487	463 (18.6)	1201	147 (12.2)	−4.9 (−7.7 to −2.1)	−8.1 (−12.1 to −4.0)
Colorectal	955	85 (8.9)	1229	100 (8.1)	904	45 (5.0)	−0.3 (−3.0 to 2.4)	−4.3 (−7.7 to −1.0)
Prostate	1793	1036 (57.8)	1364	741 (54.3)	540	229 (42.4)	−4.1 (−17.3 to 9.1)	−14.3 (−25.2 to −3.5)
Medication use								
Antibiotic for acute upper respiratory tract infection	13 917	3159 (22.7)	2560	678 (26.5)	599	136 (22.7)	1.3 (−0.5 to 3.1)	−5.1 (−6.3 to −3.8)
Antibiotic for influenza	6161	696 (11.3)	1286	183 (14.2)	348	48 (13.8)	−0.3 (−1.1 to 0.5)	−2.5 (−6.3 to 1.2)
Benzodiazepine for depression	9612	1576 (16.4)	5355	1280 (23.9)	2502	726 (29.0)	4.5 (2.5 to 6.4)	8.1 (6.3 to 9.8)
Opioid for back pain	5669	130 (2.3)	1714	77 (4.5)	750	29 (3.9)	4.5 (3.5 to 5.5)	6.7 (6.5 to 6.9)
Opioid for headache	11 515	1071 (9.3)	4618	1011 (21.9)	1704	506 (29.7)	0.7 (−1.0 to 2.4)	−1.0 (−1.4 to −0.7)
NSAID for hypertension, heart failure, or kidney disease	14 136	1470 (10.4)	6627	1040 (15.7)	2700	421 (15.6)	4.7 (3.7 to 5.7)	3.4 (1.1 to 5.7)
Imaging use								
MRI or CT for back pain	11 515	737 (6.4)	4618	471 (10.2)	1704	208 (12.2)	2.6 (−0.1 to 5.4)	5.8 (3.3 to 8.3)
Radiography for back pain	11 515	1393 (12.1)	4618	637 (13.8)	1704	269 (15.8)	2.1 (1.3 to 2.9)	4.3 (3.7 to 4.9)
MRI or CT for headache	5669	255 (4.5)	1714	93 (5.4)	750	41 (5.5)	0.1 (−1.0 to 1.3)	−0.4 (−2.4 to 1.5)

Abbreviations: CT, computed tomography; MRI, magnetic resonance imaging; NSAID, nonsteroidal anti-inflammatory drug.

^a^ Functional disability was measured using 6 questions assessing difficulties and was categorized into 3 levels: no (0 difficulties), moderate (1-2 difficulties), and severe (≥3 difficulties).

^b^ Survey weights were used to generate nationally representative estimates of adults from the 2013 to 2021 Medical Expenditure Panel Survey.

^c^ Logistic regression models were used to estimate adjusted differences in the outcomes, controlling for individual-level characteristics and year fixed effects.

Conversely, adults with moderate and severe functional disabilities were less likely to receive other low-value services, such as cervical cancer screening (adjusted differences: −4.9 [95% CI, −7.7 to −2.1] percentage points and −8.1 [95% CI, −12.1 to −4.0] percentage points, respectively), colorectal cancer screening (adjusted differences: −4.3 [95% CI, −7.7 to −1.0] percentage points for those with severe functional disabilities), prostate cancer screening (adjusted differences: −14.3 [95% CI, −25.2 to −3.5] percentage points for those with severe functional disabilities), antibiotic for acute upper respiratory tract infection (adjusted differences: −5.1 [95% CI, −6.3 to −3.8] percentage points for those with severe functional disabilities), and opioid for headache (adjusted differences: −1.0 [95% CI, −1.4 to −0.7] percentage points for those with severe functional disabilities) (Table 3).

Low-value services that could be provided during an appointment, such as benzodiazepine for depression and opioid for back pain, were more frequently used by adults with moderate and severe functional disabilities. However, services requiring a separate appointment, such as cervical cancer screening, were less frequently used.

## Discussion

Findings of this study indicate that adults with self-reported functional disabilities had higher rates of outpatient visits and prescription drug fills, which aligns with prior research reporting higher health care utilization among individuals with disabilities.^4,5,6,7,8^ Additionally, consistent with prior research, the present study found higher rates of unmet needs for medical care among those with functional disabilities.^38^ This finding suggests that greater utilization does not necessarily indicate better quality of care or higher patient satisfaction.^10^ Therefore, it is crucial to rigorously assess which specific health care services are associated with greater utilization and to identify the essential services currently underused in addressing the unmet medical needs of adults with functional disabilities.

Individuals with disabilities, who interact more frequently with the health care system, may have greater opportunities to receive both low- and high-value services; however, the findings indicate that the utilization of high- and low-value care was not consistently higher (or lower) among adults with functional disabilities. This inconsistent pattern of specific service utilization aligns with results of prior research examining the use of high- and low-value clinical services by adults with cognitive impairment, another potentially clinically and socioeconomically vulnerable patient population.^39^ We observed that both high- and low-value services provided or accessible during routine visits, such as certain diagnostic tests (eg, blood tests) and medications, were used more frequently, whereas less accessible high- and low-value services that often require separate appointments, such as cancer screenings, were used less often. Prior research indicates that the setting in which services are provided is an important factor in health care utilization.^40^ These patterns suggest that accessibility of clinical services likely plays a critical role in utilization, a highly relevant factor for adults with functional disabilities who may be more susceptible to service features that affect access.

The findings have important policy implications. In 2024, the Agency for Healthcare Research and Quality recognized the limited evidence regarding the delivery of preventive services to people with disabilities and proposed to the US Congress guiding principles for addressing barriers to service delivery.^41^ This study provides timely and nuanced evidence that the delivery of clinical services, whether high or low value, is associated with the ease of clinician prescribing and/or the effort necessary from patients to receive the services. Thus, policies focused on improving health care access (eg, workforce expansion and increases in insurance coverage) may not sufficiently address the accessibility issue. Past evidence suggests that well-intended policy interventions to promote the use of high-value services, such as eliminating cost-sharing for primary care visits, can unintentionally increase the use of office-based, low-value services.^42^

By 2030, the Centers for Medicare and Medicaid Services aims to have all Medicare beneficiaries and most Medicaid beneficiaries enrolled in accountable, value-based care programs,^43^ highlighting the urgent need for effective policy development. Given that individuals with functional disabilities often have unique needs, tailored strategies are essential to improve the quality and efficiency of care for this large and clinically and socioeconomically vulnerable population. Since both patient and clinician factors play a role in the use of these services, policies that simultaneously engage both groups are likely to be most effective.^44,45^ To achieve optimal benefits, these efforts should be coupled with interventions that encourage the use of high-value services—especially services that require more patient effort—and that discourage the use of easily accessible but low-value care. One potential solution is to expand coverage for care coordinators, such as patient navigators and case managers, to help individuals with functional disabilities in scheduling appointments and addressing transportation and mobility challenges.^46,47^ Policies could build on the 4 new *Current Procedural Terminology* codes in the 2024 Medicare Physician Fee Schedule, which allow health care practitioners to bill for patient navigation services offered to individuals with a cancer diagnosis.^48^

### Limitations

This study has several limitations. First, the sample was limited to noninstitutionalized US adults, limiting the generalizability of the findings to this population. Second, we used previously validated measures of functional disability, but these measures were self-reported, which may lead respondents to underreport health problems. Third, we selected specific high- and low-value services, and thus the findings may not generalize to other services in these categories. Fourth, since some measures of high- and low-value services were self-reported, there is a risk of reporting errors in the data. Fifth, we had limited ability to identify all relevant exclusions for low-value care measurement. The MEPS reports health conditions using 3-digit *International Classification of Diseases* diagnosis and procedure codes, which may restrict our capacity to identify competing diagnoses or exclude conditions linked to clinical red flags. Sixth, while we adjusted for differences in sample characteristics, residual differences in individual-level characteristics may still exist.

## Conclusions

In this cross-sectional study, we found that higher overall health care utilization in adults with functional disabilities did not imply that their medical needs were fully met or that higher aggregate use necessarily led to increased use of evidence-based care. Ease of access to services played a critical role in the use of both high- or low-value care among adults with functional disabilities, independent of clinical value. This finding underscores the need for health policies that go beyond merely increasing access to clinician visits or prescription drugs. Instead, health policies should focus on the development and implementation of nuanced strategies to enhance use of recommended evidence-based services—particularly those that are challenging to access by adults with functional disabilities—while reducing reliance on easily accessible but low-value care.

## Notes

Supplement 1.
eMethods
**eTable 1.** Measures for High- and Low-Value Care **eTable 2.** Chronic Conditions in MEPS

Supplement 2.
Data Sharing Statement

## References

[1] Centers for Disease Control and Prevention. Disability impacts all of us. Accessed October 11, 2024. https://www.cdc.gov/ncbddd/disabilityandhealth/infographic-disability-impacts-all.html

[2] Williams JS, Egede LE. The association between multimorbidity and quality of life, health status and functional disability. Am J Med Sci. 2016;352(1):45-52. doi:10.1016/j.amjms.2016.03.004 27432034

[3] Krahn GL, Walker DK, Correa-De-Araujo R. Persons with disabilities as an unrecognized health disparity population. Am J Public Health. 2015;105(suppl 2):S198-S206. doi:10.2105/AJPH.2014.302182 25689212 PMC4355692

[4] Park S, Stimpson JP. Health care expenses and financial hardship among Medicare beneficiaries with functional disability. JAMA Netw Open. 2024;7(6):e2417300. doi:10.1001/jamanetworkopen.2024.17300 38884997 PMC11184460

[5] Mitra S, Findley PA, Sambamoorthi U. Health care expenditures of living with a disability: total expenditures, out-of-pocket expenses, and burden, 1996 to 2004. Arch Phys Med Rehabil. 2009;90(9):1532-1540. doi:10.1016/j.apmr.2009.02.020 19735781

[6] Khavjou OA, Anderson WL, Honeycutt AA, . National health care expenditures associated with disability. Med Care. 2020;58(9):826-832. doi:10.1097/MLR.0000000000001371 32826747 PMC7505687

[7] Chan L, Beaver S, Maclehose RF, Jha A, Maciejewski M, Doctor JN. Disability and health care costs in the Medicare population. Arch Phys Med Rehabil. 2002;83(9):1196-1201. doi:10.1053/apmr.2002.34811 12235597

[8] Fried TR, Bradley EH, Williams CS, Tinetti ME. Functional disability and health care expenditures for older persons. Arch Intern Med. 2001;161(21):2602-2607. doi:10.1001/archinte.161.21.2602 11718592

[9] Ankuda CK, Ornstein KA, Kelley AS. Assessing health care use trajectories after the onset of functional disability: application of a group-based trajectory model. J Gerontol B Psychol Sci Soc Sci. 2022;77(suppl 1):S31-S38. doi:10.1093/geronb/gbab233 35034108 PMC9122631

[10] Grady D, Redberg RF. Less is more: how less health care can result in better health. Arch Intern Med. 2010;170(9):749-750. doi:10.1001/archinternmed.2010.90 20458080

[11] Shrank WH, Rogstad TL, Parekh N. Waste in the US health care system: estimated costs and potential for savings. JAMA. 2019;322(15):1501-1509. doi:10.1001/jama.2019.13978 31589283

[12] Schpero WL, Morden NE, Sequist TD, Rosenthal MB, Gottlieb DJ, Colla CH. For selected services, Blacks and Hispanics more likely to receive low-value care than Whites. Health Aff (Millwood). 2017;36(6):1065-1069. doi:10.1377/hlthaff.2016.1416 28583965 PMC5568010

[13] Park S, Wadhera RK. Use of high- and low-value health care among US adults, by income, 2010-19. Health Aff (Millwood). 2024;43(7):1021-1031. doi:10.1377/hlthaff.2023.00661 38950294

[14] Park S, Nguyen AM. Use of high- and low-value care among US adults by education levels. Fam Pract. 2023;40(4):560-563. doi:10.1093/fampra/cmad082 37543851

[15] Park S, Stimpson JP. Unmet need for medical care among Medicare beneficiaries by health insurance literacy and disability. Disabil Health J. 2024;17(2):101548. doi:10.1016/j.dhjo.2023.101548 37980229

[16] Agency for Healthcare Research and Quality. Medical Expenditure Panel Survey: data overview. Accessed October 11, 2024. https://www.meps.ahrq.gov/mepsweb/data_stats/data_overview.jsp

[17] Park S, Stimpson JP. Effects of Medicare eligibility at age 65 among individuals with and without functional disability. J Gen Intern Med. Published online October 4, 2024. doi:10.1007/s11606-024-09060-7 39367286 PMC11968645

[18] Centers for Disease Control and Prevention. Disability datasets: population surveys that include the standard disability questions. Accessed October 11, 2024. https://www.cdc.gov/ncbddd/disabilityandhealth/datasets.html

[19] U.S. Department of Health and Human Services. HHS implementation guidance on data collection standards for race, ethnicity, sex, primary language, and disability status. Accessed October 11, 2024. https://www.medicaid.gov/sites/default/files/2023-12/arp-sec9817-overview-infographic_0.pdf

[20] Levine DM, Linder JA, Landon BE. The quality of outpatient care delivered to adults in the United States, 2002 to 2013. JAMA Intern Med. 2016;176(12):1778-1790. doi:10.1001/jamainternmed.2016.6217 27749962

[21] Levine DM, Landon BE, Linder JA. Quality and experience of outpatient care in the United States for adults with or without primary care. JAMA Intern Med. 2019;179(3):363-372. doi:10.1001/jamainternmed.2018.6716 30688977 PMC6439688

[22] Park S, Jung J, Burke RE, Larson EB. Trends in use of low-value care in traditional fee-for-service Medicare and Medicare Advantage. JAMA Netw Open. 2021;4(3):e211762. doi:10.1001/jamanetworkopen.2021.1762 33729504 PMC7970337

[23] Park S, Wadhera RK, Jung J. Effects of Medicare eligibility and enrollment at age 65 years on the use of high-value and low-value care. Health Serv Res. 2023;58(1):174-185. doi:10.1111/1475-6773.14065 36106508 PMC9836961

[24] Siu AL; U.S. Preventive Services Task Force. Screening for breast cancer: U.S. Preventive Services Task Force recommendation statement. Ann Intern Med. 2016;164(4):279-296. doi:10.7326/M15-2886 26757170

[25] Vesco KK, Whitlock EP, Eder M, . *Screening for Cervical Cancer: A Systematic Evidence Review for the US Preventive Services Task Force—Report No.: 11-05156-EF-1*. Agency for Healthcare Research and Quality; 2011. Accessed October 11, 2024. https://www.ncbi.nlm.nih.gov/books/NBK66099/22132428

[26] Whitlock EP, Lin J, Liles E, . *Screening for Colorectal Cancer: An Updated Systematic Review—Evidence Syntheses, No. 65.1. Report No.: 08-05-05124-EF-1*. Agency for Healthcare Research and Quality; 2008. Accessed October 11, 2024. https://www.ncbi.nlm.nih.gov/books/NBK35179/

[27] Piper MA, Evans CV, Burda BU, . *Screening for High Blood Pressure In Adults: A Systematic Evidence Review for the US Preventive Services Task Force*. Agency for Healthcare Research and Quality; 2014. Accessed October 11, 2024. https://www.ncbi.nlm.nih.gov/books/NBK35179/pdf/Bookshelf_NBK35179.pdf 25632496

[28] Helfand M, Carson S. *Screening for Lipid Disorders in Adults: Selective Update of 2001 US Preventive Services Task Force Review.* Agency for Healthcare Research and Quality; 2008. Accessed October 11, 2024. https://www.ncbi.nlm.nih.gov/books/NBK33494/20722146

[29] Grohskopf LA, Alyanak E, Broder KR, . Prevention and control of seasonal influenza with vaccines: recommendations of the advisory committee on immunization practices - United States, 2020-21 influenza season. MMWR Recomm Rep. 2020;69(8):1-24. doi:10.15585/mmwr.rr6908a1 32820746 PMC7439976

[30] American Diabetes Association. Standards of medical care in diabetes-2016 abridged for primary care providers. Clin Diabetes. 2016;34(1):3-21. doi:10.2337/diaclin.34.1.3 26807004 PMC4714725

[31] Fenton JJ, Weyrich MS, Durbin S, Liu Y, Bang H, Melnikow J. Prostate-specific antigen-based screening for prostate cancer: evidence report and systematic review for the US Preventive Services Task Force. JAMA. 2018;319(18):1914-1931. doi:10.1001/jama.2018.3712 29801018

[32] Cooper RJ, Hoffman JR, Bartlett JG, ; American Academy of Family Physicians; American College of Physicians-American Society of Internal Medicine; Centers for Disease Control and Prevention. Principles of appropriate antibiotic use for acute pharyngitis in adults: background. Ann Intern Med. 2001;134(6):509-517. doi:10.7326/0003-4819-134-6-200103200-00019 11255530

[33] Harris AM, Hicks LA, Qaseem A; High Value Care Task Force of the American College of Physicians and for the Centers for Disease Control and Prevention. Appropriate antibiotic use for acute respiratory tract infection in adults: advice for high-value care from the American College of Physicians and the Centers for Disease Control and Prevention. Ann Intern Med. 2016;164(6):425-434. doi:10.7326/M15-1840 26785402

[34] Trangle M, Gursky J, Haight R, . Adult depression in primary care. Institute for Clinical Systems Improvement. Accessed October 11, 2024. https://www.icsi.org/guideline/depression/

[35] American Society of Anesthesiologists. Choosing Wisely: pain medicine. Accessed October 11, 2024. https://www.choosingwisely.org/societies/american-society-of-anesthesiologists-pain-medicine/

[36] American College of Radiology. Choosing Wisely: don’t do imaging for uncomplicated headache. Accessed October 11, 2024. https://www.aafp.org/pubs/afp/collections/choosing-wisely/38.html

[37] Chou R, Fu R, Carrino JA, Deyo RA. Imaging strategies for low-back pain: systematic review and meta-analysis. Lancet. 2009;373(9662):463-472. doi:10.1016/S0140-6736(09)60172-0 19200918

[38] Park S, Stimpson JP. Unmet need for medical care among fee-for-service Medicare beneficiaries with high and low need. J Gen Intern Med. 2023;38(9):2059-2068. doi:10.1007/s11606-023-08145-z 37095329 PMC10361899

[39] Barthold D, Jiang S, Basu A, . Utilization of low- and high-value health care by individuals with and without cognitive impairment. Am J Manag Care. 2024;30(7):316-323. doi:10.37765/ajmc.2024.89580 38995830 PMC11429857

[40] Mafi JN, Wee CC, Davis RB, Landon BE. Association of primary care practice location and ownership with the provision of low-value care in the United States. JAMA Intern Med. 2017;177(6):838-845. doi:10.1001/jamainternmed.2017.0410 28395013 PMC5540052

[41] Weinick RM, Sessums LL, Boicourt RM. *Research to Improve the Delivery of Clinical Preventive Services to People With Disabilities*. Agency for Healthcare Research and Quality; 2024. Accessed October 11, 2024. https://www.ahrq.gov/sites/default/files/wysiwyg/prevention/stakeholder-report_0824.pdf

[42] Cliff BQ, Hirth RA, Mark Fendrick A. Spillover effects from a consumer-based intervention to increase high-value preventive care. Health Aff (Millwood). 2019;38(3):448-455. doi:10.1377/hlthaff.2018.05015 30830812 PMC8168457

[43] Fowler L, Rawal P, Fogler S, Waldersen B, O’Connell M, Quinton J. The CMS Innovation Center’s strategy to support person-centered, value-based specialty care. Centers for Medicare & Medicaid Services. Accessed October 11, 2024. https://www.cms.gov/blog/cms-innovation-centers-strategy-support-person-centered-value-based-specialty-care

[44] Colla CH, Mainor AJ, Hargreaves C, Sequist T, Morden N. Interventions aimed at reducing use of low-value health services: a systematic review. Med Care Res Rev. 2017;74(5):507-550. doi:10.1177/1077558716656970 27402662

[45] Sypes EE, de Grood C, Clement FM, . Understanding the public’s role in reducing low-value care: a scoping review. Implement Sci. 2020;15(1):20. doi:10.1186/s13012-020-00986-0 32264926 PMC7137456

[46] Freund KM, Battaglia TA, Calhoun E, ; Writing Group of the Patient Navigation Research Program. Impact of patient navigation on timely cancer care: the Patient Navigation Research Program. J Natl Cancer Inst. 2014;106(6):dju115. doi:10.1093/jnci/dju115 24938303 PMC4072900

[47] Ruiz S, Giuriceo K, Caldwell J, Snyder LP, Putnam M. Care coordination models improve quality of care for adults aging with intellectual and developmental disabilities. J Disabil Policy Stud. 2020;30(4):191-201. doi:10.1177/1044207319835195

[48] Centers for Medicare & Medicaid Services. Medicare and Medicaid programs; CY 2024 payment policies under the physician fee schedule and other changes to Part B payment and coverage policies; Medicare Shared Savings Program requirements; Medicare Advantage; Medicare and Medicaid provider and supplier enrollment policies; and basic health program final rule. Accessed October 11, 2024. https://public-inspection.federalregister.gov/2023-24184.pdf

