# Supplementary material for: Health Care Utilization Patterns Among Adults With or Without Functional Disabilities
Source: JAMA Netw Open. 2025 Apr 11;8(4):e254729. doi: 10.1001/jamanetworkopen.2025.4729 (PMC11992608; doi:10.1001/jamanetworkopen.2025.4729)
Supplement: Supplement 1. — eMethods eTable 1. Measures for High- and Low-Value Care eTable 2. Chronic Conditions in MEPS [file jamanetwopen-e254729-s001.pdf]

Supplemental Online Content

Park S, Stimpson JP, Fendrick AM. Health care utilization patterns among adults with or without functional disabilities. *JAMA Netw Open*. Published online April 11, 2025.
doi:10.1001/jamanetworkopen.2025.4729

eMethods

eTable 1. Measures for high- and low-value care

eTable 2. Chronic Conditions in MEPS

This supplemental material has been provided by the authors to give readers additional information about their work.

© 2025 Park S et al. *JAMA Network Open*

eMethods

Data from MEPS

MEPS collects data from two primary sources. The Household Component (HC) collects data from individual household members through survey questionnaires and the Medical Provider Component (MPC) collects data from a sample of health care providers to MEPS HC respondents. The HC data includes demographic, socioeconomic, and health characteristics. The MPC data includes dates of visits or services, types of health care services used, and diagnoses codes for medical encounters. For this study, we used five datasets from MEPS: the full-year consolidated data files from the HC, outpatient visits files, office-based medical provider visit files, prescribed medicine files, and medical conditions files from the MPC.

Measures for functional disability

Functional disability was defined based on six questions assessing difficulties in seeing, hearing, memory/concentration, walking, self-care, and performing errands due to a physical, mental, or emotional condition. These six questions adhere to the established data standard for survey questions on disability codified by the US Department of Health and Human Services and serve as a fundamental measure. These following questions from MEPS included:

- "Does anyone in the family have any difficulty seeing?"
- "Does anyone in the family have any difficulty hearing?"
- "Do any of the adults in the family experience confusion or memory loss such that it interferes with daily activities?"
- "Does anyone in the family have any difficulty walking, climbing stairs, grasping objects, reaching overhead, lifting, bending or stooping, or standing for long periods?"

© 2025 Park S et al. *JAMA Network Open*

- “Does anyone in the family receive help or supervision with personal care such as bathing, dressing, or getting around the house?”
- “Because of a physical, mental, or emotional condition, do you have difficulty doing errands alone such as visiting a doctor's office or shopping?”

If the response was “yes”, a follow-up question was asked to determine which household member(s) had difficulty. Based on this information, we assigned a value of 1 to indicate the presence of a difficulty if it was attributed to the respondent and a value of 0 to indicate no difficulty. We assigned a value of 1 to indicate the presence of a difficulty and 0 to indicate none. The scores from the six questions were then aggregated. Functional disability was classified into three levels: none (no difficulties in any of the six questions), moderate (1-2 difficulties), and severe (≥3 difficulties).

Measurement for health care utilization and unmet need for medical care

Health care utilization (from the MPC data)

- Inpatient, outpatient, emergency room, and prescription drugs: we used event-level data (e.g., claims data) to measure health care utilization.

Unmet need for medical care (from the HC data)

The MEPS asked questions at the family level, but also asked which family member was the case. Therefore, we only coded "yes" for the family member who was part of our study sample.

- Experiencing delays in obtaining necessary medical care: “in the last 12 months, was anyone in the family delayed in getting medical care, tests, or treatment they or a doctor believe necessary?”

© 2025 Park S et al. *JAMA Network Open*

- Being unable to get medical care: “in the last 12 months, was anyone in the family unable to obtain medical care, tests, or treatment they or a doctor believe necessary?”

Low-value cancer screening

For several services, evidence has demonstrated high value for some age groups but not others. Notably, screening for cervical, colorectal, and prostate cancers have been found to be low value for elderly adults. Therefore, for the analysis of low-value service use among elderly adults, we included binary measures of these three additional low-value services.

© 2025 Park S et al. *JAMA Network Open*

eTable 1. Measures for high- and low-value care

Measure	MEPS data source	Measure (numerator)	Eligible population (denominator)
High-value care			
Cancer screening			
Breast cancer screening	Self-report	Mammogram within past 2 years	Women aged between 50 and 74 years
Cervical cancer screening	Self-report	Papanicolaou test within past 3 years	Women aged 21-65
Colorectal cancer screening	Self-report	Colonoscopy within past 10 years, sigmoidoscopy within past 5 years, or hemoccult test within past year	Individuals aged between 50 and 75 years
Diagnostic and preventive testing			
Blood pressure measurement	Self-report	Blood pressure measurement within 2 years	All individuals
Cholesterol measurement	Self-report	Cholesterol measurement within any interval	Individuals older than 35 years
Influenza vaccine	Self-report	Influenza vaccine within 1 year	Individuals older than 50 years
Diabetes care			
HgA1c measurement	Diabetes care survey	HgA1c measurement at least twice yearly	Individuals with diabetes
Foot examination	Diabetes care survey	Food examination within 1 year	Individuals with diabetes
Eye examination	Diabetes care survey	Retinal examination within 1 year	Individuals with diabetes
Low-value care			
Antibiotic use			
Antibiotics for acute upper respiratory infection	Prescribed medicine	Antibiotic prescription during visit	Individuals with a primary diagnosis of acute upper respiratory infection without a diagnosis of bacterial infection, chronic obstructive pulmonary disease, or cancer in the given survey year (as competing diagnosis for acute upper respiratory infection)

© 2025 Park S et al. *JAMA Network Open*

Antibiotics for influenza	Prescribed medicine	Antibiotic prescription during visit	Individuals with a primary diagnosis of influenza without a diagnosis of bacterial infection, chronic obstructive pulmonary disease, or cancer in the given survey year
Medications	Prescribed medicine		
Benzodiazepine for depression	Prescribed medicine	Benzodiazepine prescription during visit	Individuals with a diagnosis of depression
Opioid for back pain	Prescribed medicine	Opioid prescription during visit	Individuals with a diagnosis of back pain but no diagnosis of fever or cancer in the give survey year
Opioid for headache	Prescribed medicine		
NSAID use for hypertension, heart failure, or kidney disease	Prescribed medicine	NSAID prescription during visit	Individuals with a diagnosis of hypertension, hear failure, or kidney disease in the give survey year
Imaging			
MRI/CT for back pain	Office-based and outpatient	MRI or CT scan during visit	Individuals with a diagnosis of back pain but no diagnosis of fever or cancer in the given survey year
Radiograph for back pain	Office-based and outpatient	Radiograph during visit	Individuals with a diagnosis of back pain but no diagnosis of fever or cancer in the given survey year
MRI/CT for headache	Office-based and outpatient	MRI or CT scan during visit	Individuals with a diagnosis of headache but no diagnosis of pregnancy, cancer, or epilepsy in the given survey year
Cancer screening			
Cervical cancer screening	Self-report	Papanicolaou test in the given survey year	Women older than 65 years without a diagnosis of cervical cancer or other genital cancer found among women in the prior year
Colorectal cancer screening	Self-report	Colonoscopy within past 10 years, sigmoidoscopy within past 5 years, or hemoccult test within past year	Individuals older than 80 years without a diagnosis of colon cancer in the prior year

© 2025 Park S et al. *JAMA Network Open*

Prostate cancer screening	Self-report	Prostate-specific antigen test in the given survey year	Men older than 70 years without a diagnosis of prostate cancer in the prior year

Abbreviation: MEPS, Medical Expenditure Panel Survey; NSAID, nonsteroidal anti-inflammatory drug; MRI, magnetic resonance imaging; CT, computed tomography.

© 2025 Park S et al. *JAMA Network Open*

eTable 2. Chronic Conditions in MEPS

Chronic Conditions
Arthritis
Asthma
Autism
Cancer
Cardiac arrhythmias
Chronic kidney disease
Chronic obstructive pulmonary disease
Congestive heart failure
Coronary artery disease
Dementia
Depression
Diabetes
Hepatitis
HIV
Hyperlipidemia
Hypertension
Osteoporosis
Schizophrenia
Stroke
Substance use disorders
