# Supplementary material for: Health Care Utilization Patterns Among Adults With or Without Functional Disabilities
Source: JAMA Netw Open. 2025 Apr 11;8(4):e254729. doi: 10.1001/jamanetworkopen.2025.4729 (PMC11992608; doi:10.1001/jamanetworkopen.2025.4729)
Supplement: Supplement 2. — Data Sharing Statement [file jamanetwopen-e254729-s002.pdf]

Data Sharing Statement

Data
Data available: No

Additional Information
Explanation for why data not available: The data is publicly available; however, we are happy to provide our specific datasets upon request.
